# Neuroleptic Malignant Syndrome in a Patient with Tongue Cancer: A Report of a Rare Case

**DOI:** 10.1155/2013/542130

**Published:** 2013-06-18

**Authors:** Osamu Baba, Kenji Yamagata, Yasushi Tomidokoro, Akira Tamaoka, Hiroyuki Itoh, Toru Yanagawa, Kojiro Onizawa, Hiroki Bukawa

**Affiliations:** ^1^Department of Oral and Maxillofacial Surgery, Faculty of Medicine, University of Tsukuba, 1-1-1 Tennodai, Ibaraki, Tsukuba City 305-8575, Japan; ^2^Department of Clinical Pathophysiology of the Neurological Disorders, Faculty of Medicine, University of Tsukuba, Japan

## Abstract

*Background*. Neuroleptic malignant syndrome (NMS) is a rare but life-threatening complication of neuroleptic drugs, which are used widely in head and neck cancer (HANC) patients who develop delirium. *Methods and Results*. Postoperative delirium in a 39-year-old man with tongue cancer was treated with haloperidol and chlorpromazine. Three days after the first administration of antipsychotics, the patient exhibited elevated body temperature, autonomic and extrapyramidal symptoms, and impaired consciousness. A definitive diagnosis was made using the research diagnostic criteria for NMS in the DSM-IV, and the antipsychotics were immediately discontinued. The patient was given dantrolene and bromocriptine to treat the NMS. The patient's hyperthermia, elevated creatinin kinase (CK), and muscle rigidity improved gradually, with all symptoms of NMS resolving completely by 13 days after the diagnosis. *Conclusions*. HANC surgeons must be alert for early signs of NMS and use antipsychotics conservatively to avoid NMS and its potentially fatal outcome.

## 1. Introduction

Neuroleptic malignant syndrome (NMS) is a rare but life-threatening disorder caused by an adverse reaction to neuroleptic (antipsychotic) drugs and characterized by hyperthermia, severe muscle rigidity, and changes in autonomic and mental status [1]. Most cases of clinical NMS have been reported by psychiatrists; however, NMS can occur in any setting in which psychotropic drugs are administered. In nonpsychiatric cases treated with neuroleptics, NMS can be difficult to diagnose, particularly because the symptoms are consistent with other, more expected, disorders. However, early diagnosis is extremely important to reduce the risk of death. The frequency of the syndrome ranges from 0.07 to 2.2% in patients treated with neuroleptic drugs, and the mortality is 10 to 30% [2, 3]. Although the frequency of NMS among patients taking neuroleptics has decreased, it remains a significant source of morbidity and mortality in this patient group [4]. To our knowledge, a few reports have been published about NMS in patients with head and neck cancer (HANC) [5, 6]. We report a rare case of NMS that occurred postoperatively in a tongue cancer patient whose surgery consisted of hemiglossectomy, neck dissection, and immediate reconstruction.

## 2. Case Report

A 39-year-old man came to the Department of Oral and Maxillofacial Surgery, University of Tsukuba Hospital, complaining of a painful mass of the tongue border. His medical, social, and family histories were unremarkable. The diagnosis was tongue cancer (T2N1 M0). He received induction chemotherapy of oral fluoropyrimidine, S-1 at 120 mg/day for 2 weeks. After the induction chemotherapy, hemiglossectomy, modified radical neck dissection, and immediate reconstruction with a free forearm flap were performed under general anesthesia, without any complications. The patient was sedated with intravenously administered propofol and dexmedetomidine hydrochloride. Anesthesia was induced and maintained with sevoflurane, propofol, and dexmedetomidine hydrochloride. The operative time was 10 hours, and the blood loss was 420 mL.

**Figure 1:**
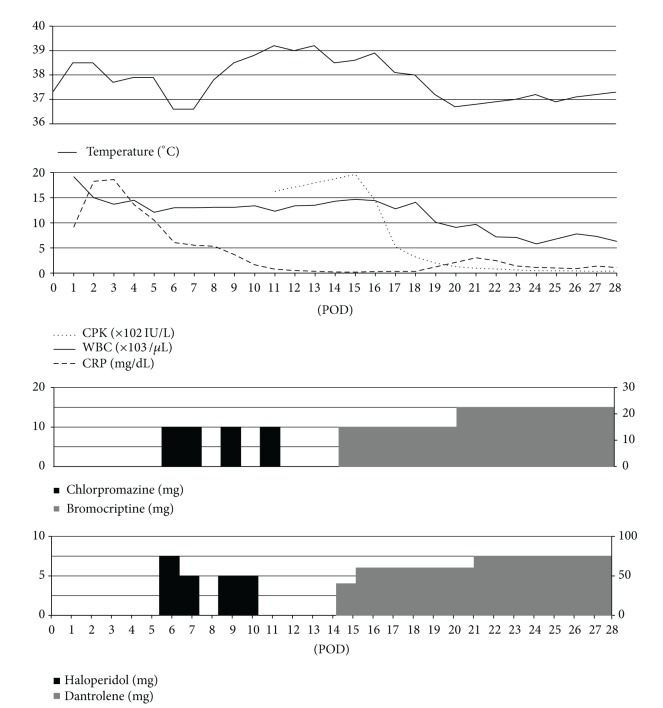
Clinical course of NMS. POD: postoperative days. On POD 15, treatment of NMS began.

The patient's postoperative clinical course is shown in Figure 1. He was sedated with propofol and dexmedetomidine hydrochloride to prevent postoperative thrombosis in the microvascular anastomosis. On postoperative day 6, the patient became severely agitated, and postoperative delirium was diagnosed. To treat the delirium, 5–10 mg of haloperidol and 10 mg of chlorpromazine were administered intravenously for 5 days. Three days after the first administration of the antipsychotic drugs, the patient's body temperature (BT) rose to over 38.5°C, and severe diaphoresis was observed. Drug-induced fever or infectious disease was suspected, and the antibacterial agent was changed from cefozopran to ciprofloxacin; however, the patient's BT continued to rise. 

**Figure 2:**
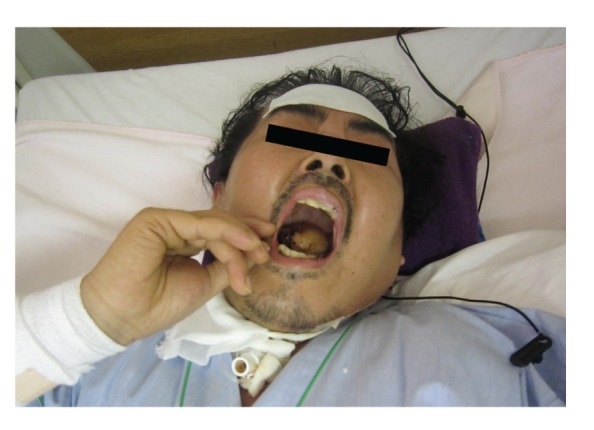
Case patient exhibiting NMS symptoms. Hyperthermia, significant extrapyramidal symptoms, various autonomic symptoms, and impaired consciousness were observed.

On the ninth day after the first use of antipsychotic drugs, the patient's hyperthermia continued, and it was accompanied by changes in consciousness level, difficulty closing the mouth, dysphagia, tremor, muscle rigidity, diaphoresis, tachycardia, and elevated blood pressure (Figure 2). The patient's conscious level was confusing. His blood pressure was 143/77 mmHg. Laboratory data on that day showed an elevated white blood cell count (WBC, 14.7 × 10^3^/*μ*L), elevated creatinin kinase (CK, 1964 U/l), and a detectable C-reactive protein (CRP) value (0.17 mg/dL). There was a significant discrepancy between the WBC and CRP. Bacterial cultures of the blood and urine were negative. Chest X-ray and brain computed tomography revealed no abnormalities. The patient was referred to the Department of Neurology, where a definitive diagnosis of NMS was made according to the diagnostic research criteria of the DSM-IV [7]. The antipsychotic medications were immediately discontinued.

To treat the NMS, 40 mg of dantrolene was given intravenously, and 15 mg of bromocriptine was delivered by nasogastric tube, 15 days after the first administration of antipsychotics. By the next day, the patient's extrapyramidal and autonomic dysfunction began to improve, and the laboratory data began to return to the normal range. The patient's BT resolved to under 37°C, and the CK level decreased to the normal range within 6 days of beginning treatment for the NMS. The patient's muscle rigidity improved gradually, and the symptoms of NMS had completely resolved by 13 days after the date of NMS diagnosis.

## 3. Discussion

Although the exact pathophysiologic mechanisms of NMS are uncertain, an acute reduction in brain dopamine activity is thought to have a primary role [4, 8–10]. NMS is probably caused by a complex interaction between the neuroleptic medication and host susceptibilities. Familial cluster of NMS and presence of a specific allele of the dopamine D2 receptor gene were reported [11, 12]. Two hypotheses have been proposed to explain the syndrome: (a) the neuroleptic medications cause a blockade of central dopamine receptors, and (b) the neuroleptic medications interact with skeletal muscle defects. In the first hypothesis, dopaminergic receptor blockade by neuroleptics may interfere with central thermoregulation. Heat is produced in response to stimulation by serotonin in the hypothalamus, and dopamine inhibits this process. A dopaminergic blockade would, therefore, reduce the inhibition of the serotonin-induced temperature increase, thus, leading to the hyperthermia seen in NMS [13, 14]. Interference with nigrostriatal dopamine pathways may lead to muscle rigidity and tremor. The second hypothesis posits that NMS shares its pathophysiology with that of malignant hyperthermia [2]. As in patients with malignant hyperthermia, an *in vitro* investigation of tissue samples from NMS patients revealed multiple defects in skeletal muscle, most of which were associated with increased calcium released from the sarcoplasmic reticulum [15].

Major symptoms of NMS include hyperthermia, tachycardia, diaphoresis, muscle rigidity, tremor, mutism, and altered consciousness. Several nonspecific laboratory abnormalities, including elevated CK and leukocytosis, are also seen [10]. The diagnostic criteria of Caroff and Mann [10] and Levenson [16] are considered valid for a definitive diagnosis. However, the above symptoms are for full-blown NMS and are rarely seen early in the disease; therefore, NMS is difficult to diagnose, especially in its early stages. Research criteria from the DSM-IV have been used for the diagnosis of NMS (Table 1) [7]. In the present case, the patient's condition met criterion A and 8 of 10 items of criterion B in the DSM-IV criteria. There were no other identifiable substances, neurologic/medical etiologies, or psychiatric disorders that might better explain the patient's symptoms (criteria C and D). Recently, a novel diagnostic criterion for NMS is published by an international multispecialty consensus group in 2011 for validation [17]. 

Most neuroleptic medications including “atypical” antipsychotic drugs, carry some risk of causing NMS. Among the typical neuroleptics, haloperidol is the most common associated with NMS, and chlorpromazine, fluphenazine, and levomepromazine are all associated with NMS onset [14]. NMS most often occurs with the initiation of neuroleptics or an increase in dosage, but it is rarely seen after the sudden discontinuation of drug therapy [18].

NMS can be particularly difficult to diagnose, in part because symptoms develop over 24 to 72 hours and can last from 1 to 44 days (about 10 days on average) [19]. Furthermore, there is no typical sequence of symptoms, whereas extrapyramidal symptoms usually occur before autonomic ones. Although mental status change can be an initial symptom of the disorder [20], it is difficult to distinguish from postoperative delirium. In the current patient's case, NMS occurred 3 days after initiation of 2 neuroleptics, haloperidol, and chlorpromazine and lasted through the subsequent 5 days during which these drugs continued to be used. This timing agrees with previous reports [18, 19]. However, it should be mentioned that NMS can occur even after a single dose or after use of for many years [21].

Psychiatric conditions such as catatonia and agitation have been substantiated in case-control studies [22]. In addition, several clinical, systemic, and metabolic factors including undernutrition, dehydration, preexisting abnormalities of the central nervous system, pharmacological and treatment variables, acute medical illness such as surgery, and a history of NMS may be risk factors for NMS, in combination with neuroleptic use [4]. In the case of dehydration, hyperthermia is exacerbated by decreased blood volume, which induces peripheral vasoconstriction and impairs heat dissipation. Other risk factors for NMS may include stress, humidity, and the concomitant use of lithium, anticholinergic agents, or some antidepressants [23]. Nearly 80% of NMS patients have been reported to be undernutrition and dehydration prior to the onset of the disease [8]. The current patient had an increased risk for NMS because of postoperative stress from his prolonged period of admission to intensive care units, dehydration from high fever (over 38.0°C), and postoperative undernutrition, because the patient did not take any oral nutrition before the administration of neuroleptics. 

NMS, a life-threatening neurological emergency, requires the immediate discontinuation of neuroleptic medications and the institution of supportive medical therapy, including body fluid volume resuscitation and general cooling [24]. Serial monitoring for hyperthermia, cardiovascular collapse, acute myoglobin-induced renal failure, respiratory failure, and aspiration pneumonia is critical [4, 9, 10]. Dantrolene, a muscle relaxant that is specifically indicated for anesthetic-induced malignant hyperthermia, is also frequently used to treat NMS [4]. Several dopaminergic agents, including bromocriptine and amantadine, have been reported to reverse parkinsonism in NMS [25, 26], reduce time to recovery, and halve mortality rates when used alone or in combination with other treatments [4]. In our case, rapid reversal of the hyperthermia and rigidity were observed within 5 days of starting treatment with dantrolene and bromocriptine. One clinical group reported that benzodiazepines and electric convulsive therapy are effective for treating NMS [4]. At present, it is difficult to compare specific treatments for NMS, because it is rare and unpredictable in its onset and progression, all of which prevent systematic investigations of treatment efficacy.

To our knowledge, only two NMS cases with HANC with the base of the tongue and the soft palate as the primary site have been reported previously [5, 6]. There are some possible explanations for the low incidence of NMS in HANC patients, in comparison with psychiatry and neurology patients. In addition to HANC surgeons not being familiar with NMS, NMS is difficult to diagnose because its symptoms are similar to those of cancer itself and sometimes to other postoperative complications like delirium. Moreover, the occurrence rate of delirium was reported to be 26.3% in patients with major HANC surgery [27]. Neuroleptic medications are widely used in clinical oncology to manage postoperative delirium. We recommend that HANC surgeons bear the possibility of NMS in mind.

In conclusion, although NMS is a rare complication in HANC patients, surgeons must be aware of the clinical features of NMS to detect its early signs and initiate prompt therapy. Furthermore, HANC surgeons should use antipsychotic drugs conservatively and with careful supervision to avoid NMS and its potential lethality.

## Figures and Tables

**Table 1:** DSM-IV diagnostic criteria of neuroleptic malignant syndrome [7].

(A) The development of severe muscle rigidity and elevated temperature associated with the use of neuroleptic medication
(B) Two (or more) of the following:
(1) diaphoresis
(2) dysphagia
(3) tremor
(4) incontinence
(5) changes in level of consciousness ranging from confusion to coma
(6) mutism
(7) tachycardia
(8) elevated or labile blood pressure
(9) leukocytosis
(10) laboratory evidence of muscle injury (e.g., elevated CPK)
(C) The symptoms in criteria A and B are not due to another substance (e.g., phencyclidine) or a neurological or other general medical conditions (e.g., viral encephalitis)
(D) The symptoms in criteria A and B are not better accounted for a mental disorder (e.g., mood disorder with catatonic features)
